# Fatal Systemic Infection Caused by Multidrug-Resistant *Clostridioides difficile* in a Domestic Rabbit: A Comprehensive Case Analysis

**DOI:** 10.3390/antibiotics15060572

**Published:** 2026-06-03

**Authors:** Vlad Iorgoni, Livia Stanga, Paula Nistor, Ioan Cristian Dreghiciu, Alexandru Gligor, Bogdan Florea, Janos Degi, Ionica Iancu, Horia Iorgoni, Cosmin Horatiu Maris, Florin Vlad, Viorel Herman

**Affiliations:** 1 Department of Infectious Diseases and Preventive Medicine, Faculty of Veterinary Medicine, University of Life Sciences “King Mihai I” from Timişoara, 300645 Timişoara, Romania; vlad.iorgoni@usvt.ro (V.I.); paula.nistor@usvt.ro (P.N.); alexandru.gligor@usvt.ro (A.G.); janosdegi@usvt.ro (J.D.); ionica.iancu@usvt.ro (I.I.); florin.vlad@usvt.ro (F.V.); viorel.herman@fmvt.ro (V.H.); 2 Discipline of Microbiology, Faculty of Medicine, “Victor Babes” University of Medicine and Pharmacy, Eftimie Murgu Square 2, 300041 Timişoara, Romania; 3 Department of Parasitology, Faculty of Veterinary Medicine, University of Life Sciences “King Mihai I” from Timişoara, 300645 Timişoara, Romania; cristian.dreghiciu@usvt.ro; 4 Department of Internal Medicine, University of Life Sciences “King Mihai I” from Timişoara, 300645 Timişoara, Romania; bogdan-alexandru.florea.fmv@usvt.ro; 5 Faculty of Physical Education and Sport, West University of Timișoara, 300223 Timișoara, Romania; horia.iorgoni@e-uvt.ro; 6 Department of Forestry, Faculty of Engineering and Applied Technologies, University of Life Sciences “King Mihai I” from Timișoara, 300645 Timișoara, Romania; cosmin.maris@usvt.ro; 7 Academy of Romanian Scientists (AOSR), Str. Ilfov, nr. 3, Sector 5, 050094 Bucharest, Romania

**Keywords:** *Clostridioides difficile*, rabbit, enterocolitis, toxigenic strains, antimicrobial resistance, gastrointestinal infection

## Abstract

**Background/Objectives**: Rabbit farming in Romania is increasingly important for providing high-quality meat, yet productivity is frequently threatened by enteric diseases, particularly in young animals. Among bacterial etiologies, *Clostridioides difficile* (*C. difficile*) has emerged as a significant gastrointestinal pathogen, with findings suggestive of systemic dissemination and public health implications. This study aimed to investigate a fatal case of *C. difficile* infection in a farmed rabbit and to characterize the pathogen’s microbiological, toxigenic, and antimicrobial profile. **Methods**: An 11-month-old male German Giant Spotted rabbit presenting acute diarrhea, anorexia, and rapid deterioration after unsupervised administration of enrofloxacin and sulfaquinoxaline was submitted postmortem. Necropsy was performed, and samples from cecum, colon, liver, spleen, mesenteric lymph nodes, lungs, and femoral bone marrow were collected. Microbiological analysis included selective culture on CCFA medium, ELISA for toxin A and B detection, MALDI-TOF MS identification, PCR confirmation, and antimicrobial susceptibility testing with the VITEK 2 system. Histopathological examination was conducted on intestinal and parenchymal tissues. **Results**: Necropsy revealed severe congestion and necrosis of the cecal and colonic mucosa, hepatomegaly, splenic congestion, and petechial hemorrhages. *C. difficile* was isolated from intestinal sites, confirmed as toxigenic by ELISA and PCR. Histopathology showed necrotizing colitis with epithelial desquamation, fibrin deposition, and heterophilic infiltration. The strain exhibited resistance to clindamycin, ampicillin, and tetracycline, with susceptibility to vancomycin, linezolid, and tigecycline. **Conclusions**: This case demonstrates that *C. difficile* can cause severe disease in rabbits, particularly following antimicrobial-induced dysbiosis. The findings underscore the importance of prudent antibiotic use, monitoring of toxigenic strains in rabbit populations, and implementation of preventive strategies to mitigate health risks in both animals and potentially humans.

## 1. Introduction

Rabbit farming has become an increasingly important branch of animal husbandry in Romania, providing a valuable source of high-quality meat with relatively low production costs. In the context of efforts to diversify protein sources and reduce meat imports, rabbits represent a complementary solution for enhancing national food security. However, the productivity of rabbit farms is frequently threatened by digestive disorders, particularly in young animals, resulting in significant economic losses [1,2,3,4,5].

One of the most concerning conditions is the enteric disease complex, a multifactorial syndrome with bacterial, viral, and nutritional etiologies. Among the bacterial causes, *Clostridium* species play a major role. Clostridial enterotoxemia, manifested by severe diarrhea, abdominal distension, and sudden death, is associated with several toxigenic species, most notably *C. perfringens*, *C. spiroforme*, *C. butyricum*, and *C. difficile* [3,5,6,7,8].

*C. difficile* is a Gram-positive, anaerobic, spore-forming, catalase-negative bacillus that produces two major toxins, Toxin A (*TcdA*) and Toxin B (*TcdB*), which represent its primary virulence factors. These exotoxins enter host cells through receptor-mediated endocytosis and require endosomal acidification for activation within the cytosol. They disrupt the actin cytoskeleton by inactivating intracellular Rho family GTPases, leading to cellular dysfunction, inflammation, and neurogenic stimulation [6,7,8,9,10,11,12].

Initially isolated in 1935 from the feces of healthy newborn infants and named *Bacillus difficilis* due to the difficulty of cultivation [13,14], *C. difficile* was long considered non-pathogenic. However, over the last three decades, it has emerged as the leading infectious cause of antibiotic-associated diarrhea in adult humans and is now recognized as one of the most important nosocomial pathogens. In severe cases, *C. difficile* infection can progress to pseudomembranous colitis, toxic megacolon, intestinal perforation, and peritonitis [11,13,14,15,16].

Recent studies have highlighted the relevance of *C. difficile* in veterinary medicine as well, with increasing reports of infections in a wide range of animal species, including pigs, cattle, horses, poultry, dogs, cats, and wild animals, as well as in food products such as meat and seafood [16,17]. The genetic similarities between human and animal isolates have raised concerns about the zoonotic potential of this pathogen and its role as a reservoir for epidemic strains [12,16,17].

In rabbits, *C. difficile* has been implicated in enterocolitis cases, particularly between 35 and 55 days of age, often associated with liquid cecal contents and severe jejunal lesions, similar to those observed in foals [18]. Experimental models have demonstrated *C. difficile* infections in rabbits following antibiotic administration or under specific pathogen-free (SPF) conditions [9,19,20,21]. However, in commercial rabbit production systems, the etiological role of *C. difficile* remains underexplored [20,21].

This study presents the case of an 11-month-old male German Giant Spotted rabbit that died following an acute episode of diarrhea, anorexia, and rapid general deterioration after unsupervised administration of enrofloxacin and sulfaquinoxaline. Necropsy performed at the Discipline of Infectious Diseases, Faculty of Veterinary Medicine in Timișoara, revealed severe congestion and necrosis of the cecal and colonic mucosa, associated with hepatomegaly, splenic congestion, and petechial hemorrhages. Samples collected from the cecum, colon, liver, spleen, mesenteric lymph nodes, lungs, and femoral bone marrow were subjected to bacteriological and histopathological examinations.

Microbiological investigations confirmed the presence of toxigenic *C. difficile*, isolated from both intestinal contents and other tissues. Identification was achieved through selective culture on CCFA medium, toxin detection by ELISA, and confirmation by MALDI-TOF MS, with reference to strain ATCC 9689. Antimicrobial susceptibility testing performed with the VITEK 2 system demonstrated resistance to several commonly used antibiotics, including clindamycin and ampicillin, while retaining susceptibility to vancomycin and linezolid.

Although *C. difficile* infection has been widely reported in rabbits and other animal species, detailed case-based investigations integrating microbiological, toxigenic, and antimicrobial susceptibility data remain limited, particularly in Romania. The present study does not aim to provide epidemiological data, but rather to offer an in-depth characterization of a clinically and pathologically well-documented case. Such reports are valuable for highlighting diagnostic approaches, identifying emerging antimicrobial resistance patterns, and improving clinical awareness in regions where data remain scarce.

The present study aims to characterize, in detail, the clinical, pathological, microbiological, and antimicrobial features of a fatal *C. difficile* infection in a domestic rabbit. The research question underlying this work is whether a comprehensive diagnostic approach, integrating culture, toxin detection, molecular confirmation, and antimicrobial susceptibility testing, can provide additional insights into disease progression and therapeutic challenges associated with this pathogen in rabbits.

## 2. Case Study

An 11-month-old male rabbit of the German Giant Spotted breed was presented postmortem to the Discipline of Infectious Diseases, Faculty of Veterinary Medicine, Timișoara, for necropsy and microbiological investigations. According to the owner’s account, prior to death the rabbit had exhibited progressive anorexia, apathy, diarrhea with mucus and occasional traces of blood, abdominal distension, and marked dehydration. The clinical course was acute, with rapid deterioration over a period of 48 h despite administration of antibiotics at home. The owner reported that the animal had received enrofloxacin and sulfaquinoxaline, drugs obtained from previous prescriptions, administered without veterinary supervision. The animal died suddenly, and the carcass was submitted immediately for pathological and microbiological evaluation.

**Figure 1:**
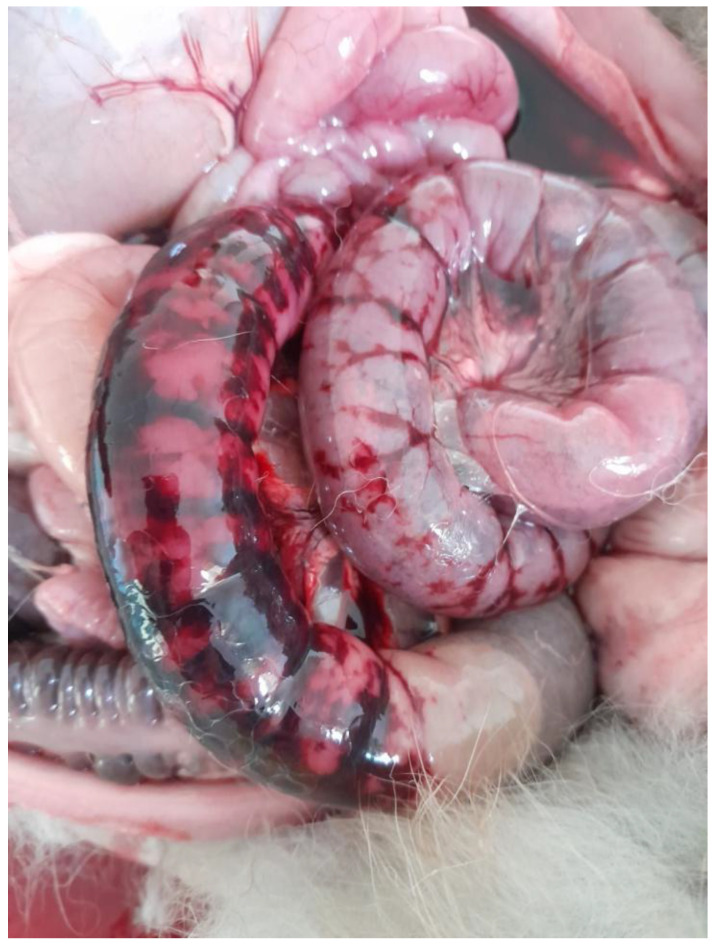
Severe intestinal congestion and hemorrhagic distension of intestinal loops.

**Figure 2:**
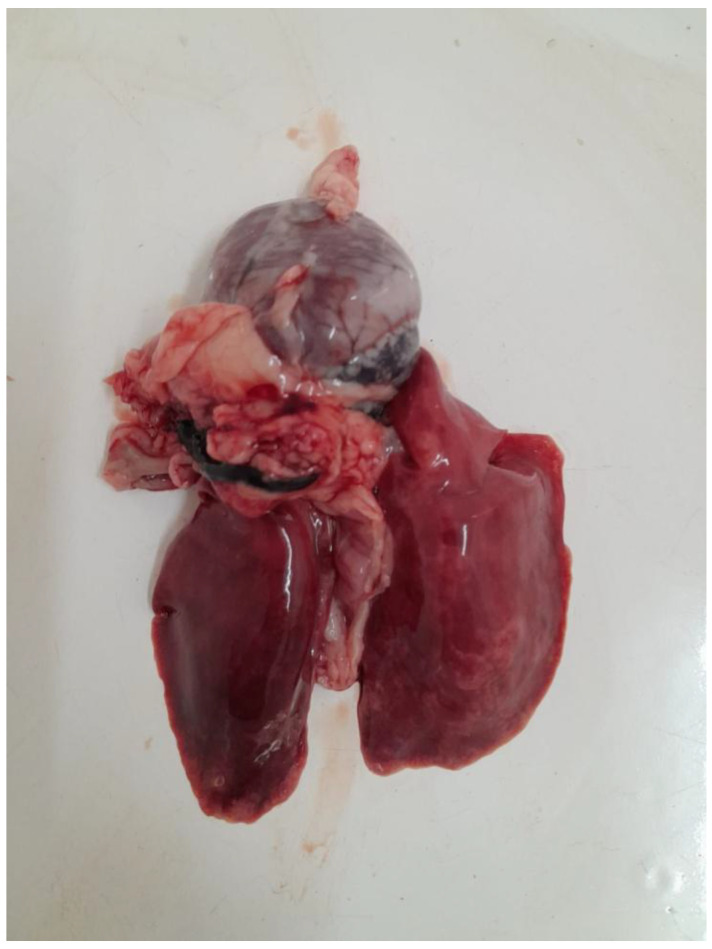
Lungs with multifocal atelectasis, consistent with circulatory disturbance and vascular damage.

At necropsy, the rabbit showed a moderate body condition score, with visibly reduced subcutaneous fat deposits, but was dehydrated, with sunken eyes, reduced fat reserves, and moderate dehydration, based on enophthalmos and decreased skin turgor. Dehydration was assessed based on enophthalmos, reduced skin elasticity, and dryness of mucous membranes. Body condition and fat reserves were evaluated based on subcutaneous and visceral fat deposits observed during necropsy. Examination of the abdominal cavity revealed severe congestion of the intestinal tract (Figure 1), particularly at the level of the cecum and colon. The cecal contents were watery, with abundant mucus and a foul odor. The mucosa of the large intestine was thickened and diffusely hyperemic and exhibited multifocal areas of necrosis and fibrinous exudation. Petechial hemorrhages were evident on the serosal surface of the cecum and colon, while mesenteric lymph nodes were enlarged and congested. The liver was moderately enlarged and friable and displayed an accentuated lobular pattern. The spleen was slightly enlarged and congested. In contrast, the stomach was moderately distended with partially digested food, and no foreign bodies were identified. Organ enlargement was assessed subjectively during necropsy and categorized descriptively as mild and moderate, based on comparison with normal anatomical references for rabbits. The lungs were congested, with multifocal areas of atelectasis, while the heart showed epicardial petechiae (Figure 2). To confirm the suspicion of systemic dissemination, bone marrow was collected aseptically from the femur immediately after opening the carcass, in addition to samples from cecum, colon, liver, spleen, and mesenteric lymph nodes.

**Figure 3:**
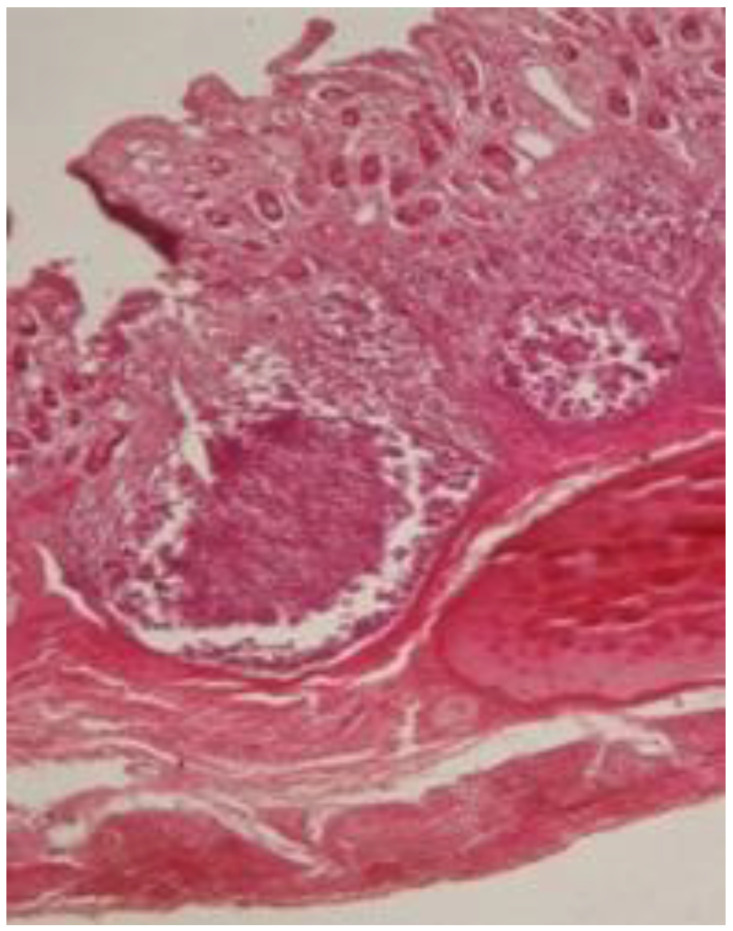
Histopathological features of enterocolitis associated with *Clostridioides difficile* infection in a rabbit. The intestinal mucosa shows villous distortion, epithelial desquamation, inflammatory infiltration in the lamina propria, and vascular congestion, with luminal cellular debris indicating active mucosal injury. H&E, scale bar = 100 µm.

**Figure 4:**
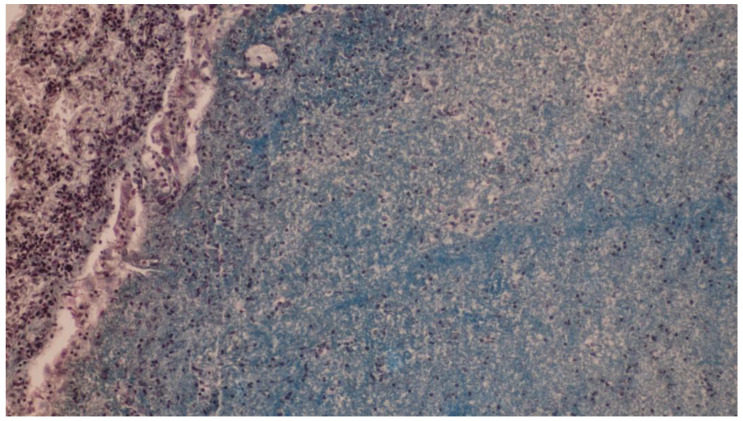
Histopathological lung changes associated with the suspicion of systemic involvement in *Clostridioides difficile* infection. Pulmonary tissue shows vascular congestion, multifocal atelectasis, mild interstitial inflammation, and focal degeneration, consistent with toxemic involvement. H&E, scale bar = 50 µm.

Tissue samples from the intestine, liver, and lungs were collected during necropsy and fixed in 10% neutral buffered formalin for 24–48 h. The samples were routinely processed, embedded in paraffin, sectioned at 4–5 µm, and stained with hematoxylin and eosin (H&E) for histopathological examination. Histopathological examination revealed severe necrotizing enterocolitis characterized by epithelial desquamation, fibrin deposition, and inflammatory cell infiltration at the intestinal level. The intestinal mucosa showed villous distortion and irregularity, multifocal epithelial desquamation, and moderate to marked inflammatory cell infiltration within the lamina propria. Vascular congestion, with dilated vessels containing erythrocytes, along with accumulation of cellular debris within the intestinal lumen and glandular structures, was consistent with active mucosal injury (Figure 3). The liver showed marked congestion and mild hepatocellular degeneration. Pulmonary lesions included vascular congestion, multifocal atelectasis, and mild interstitial inflammatory infiltration (Figure 4).

Microbiological investigation was initiated by inoculating tissue homogenates and bone marrow samples onto selective and non-selective media under anaerobic conditions. Tissue samples (cecum, colon, liver, spleen, and mesenteric lymph nodes) were aseptically collected and homogenized in sterile phosphate-buffered saline at a ratio of 1:10 using a sterile mortar and pestle. The homogenates were then centrifuged at 3000 rpm for 10 min, and the supernatant was used for bacteriological analysis.

For primary isolation of *C. difficile*, cycloserine–cefoxitin–fructose agar (CCFA) supplemented with 0.1% taurocholate was used, incubated anaerobically at 37 °C for 48 h. Parallel cultures were performed on blood agar and Schaedler anaerobe agar, providing additional growth assessment. Colonies typical of *C. difficile* appeared as greyish-white, opaque, with irregular edges and a characteristic ground-glass morphology. The colonies exhibited a distinctive horse-stable odor, which is considered suggestive of *C. difficile*. Gram staining revealed large Gram-positive bacilli with subterminal spores.

*C. difficile* was isolated from cecal and colonic contents, as well as from liver and bone marrow samples, indicating findings suggestive of systemic dissemination. No other significant bacterial pathogens were isolated in pure culture from the examined samples.

To determine the toxigenic profile, culture supernatants were subjected to enzyme-linked immunosorbent assay (ELISA) targeting toxins A and B. Both toxins were detected, confirming that the isolated strain was toxigenic. Detection of toxins A and B was performed using a commercial ELISA kit (RIDASCREEN^®^ *C. difficile* Toxin A/B, R-Biopharm, Pfungstadt, Germany), following the manufacturer’s instructions.

For definitive identification, matrix-assisted laser desorption/ionization time-of-flight mass spectrometry (MALDI-TOF MS) was employed using the Bruker Biotyper system (Billerica, MA, USA). Spectral analysis confirmed *C. difficile*, with a high log score value (>2.2), matching reference strain ATCC 9689 included in the database. PCR detection of *Clostridioides difficile* toxin genes was performed in an external accredited diagnostic laboratory using a validated real-time PCR assay (GeneXpert *C. difficile* assay, Cepheid, Sunnyvale, CA, USA), according to the manufacturer’s instructions. The assay targets the *tcdB* gene and allows qualitative detection of toxigenic strains.

ELISA testing confirmed the presence of toxins A and B in intestinal contents. PCR analysis yielded positive results for the *tcdB* gene, confirming the presence of a toxigenic *Clostridioides difficile* strain. Detection of toxins A and B was performed using a commercial ELISA kit. The intestinal content samples showed optical density (OD) values above the established cut-off, confirming the presence of both toxins. Detailed results are presented in Table 1. Positive and negative controls provided by the manufacturer were included and validated the assay.

Antimicrobial susceptibility testing was performed using the VITEK 2 Compact system with the ANC card designed for anaerobic organisms. The minimum inhibitory concentrations (MICs) were determined according to CLSI guidelines. The strain displayed resistance to clindamycin (MIC > 8 µg/mL), ampicillin (MIC > 16 µg/mL), and tetracycline (MIC > 8 µg/mL), while reduced susceptibility was noted for metronidazole (MIC 4 µg/mL). In contrast, the isolate was susceptible to vancomycin (MIC 0.5 µg/mL), linezolid (MIC 1 µg/mL), and tigecycline (MIC 0.12 µg/mL). The antibiogram confirmed the multidrug-resistant profile of the strain, which may have been selected or favored by previous antibiotic exposure. The antimicrobial susceptibility profile revealed resistance to clindamycin, ampicillin, and tetracycline, with preserved susceptibility to vancomycin, linezolid, and tigecycline (Table 2).

## 3. Discussion

The case described here illustrates the severe consequences of *C. difficile* infection in a rabbit following unsupervised antimicrobial administration. The use of enrofloxacin and sulfaquinoxaline, although not typically considered the most predisposing agents, may have disrupted the cecal microbiota sufficiently to permit the proliferation of toxigenic *C. difficile*. The presence of toxins A and B, in association with necrotic and hemorrhagic lesions of the cecum and colon, confirms the etiological role of this pathogen in the fatal outcome. The gross and histological findings align with those previously reported in antibiotic-associated colitis of rabbits, underlining the critical role of microbial balance in lagomorph gastrointestinal health [6,8,20].

The decision to focus on a single case is justified by the study design, which aims to provide a comprehensive characterization rather than a population-based analysis. In veterinary medicine, well-documented case reports remain essential for identifying emerging or underreported pathogens, particularly when associated with unusual clinical evolution, findings suggestive of systemic dissemination, or antimicrobial resistance profiles.

A noteworthy aspect of this case was the detection of *C. difficile* not only in the intestinal tract but also in extraintestinal sites, including femoral bone marrow and parenchymatous organs. This indicates a more extensive dissemination than commonly reported in rabbits, where the microorganism is usually confined to the digestive system. Such findings expand the understanding of the pathogenic potential of *C. difficile* in lagomorphs and suggest that systemic involvement may occur under certain conditions of antimicrobial disturbance and host susceptibility [6,22,23].

The antimicrobial susceptibility profile obtained with VITEK 2 revealed resistance to several antibiotics widely used in veterinary medicine, including clindamycin, ampicillin, and tetracycline, as well as reduced susceptibility to metronidazole. In contrast, susceptibility to vancomycin, linezolid, and tigecycline was preserved. These results are consistent with patterns observed in human isolates, reflecting the capacity of *C. difficile* to acquire and maintain resistance traits. The history of prior antibiotic administration in this rabbit may have facilitated the selection of resistant strains, underscoring the importance of judicious antimicrobial use in companion animals [24,25,26].

Our findings align with previous studies in other countries, including Egypt, where *C. difficile* was isolated from rabbits with enteritis and characterized using ELISA, culture, agglutination, pathogenicity testing, and PCR. Similarly to our results, a subset of isolates was confirmed to be toxigenic and correlated with clinical cases of pseudomembranous enterocolitis. These outcomes emphasize the need for systematic monitoring of *C. difficile* in commercial rabbitries and call attention to its role as a significant gastrointestinal pathogen beyond its better-known involvement in human nosocomial infections [6,8,20].

From a scientific perspective, the combined use of culture, toxin detection, MALDI-TOF MS, and PCR provides a robust diagnostic framework, ensuring high specificity and reliability of the results. This multi-step approach represents a strength of the study and aligns with current recommendations for the accurate identification of toxigenic *C. difficile* strains.

The pathogenicity of *C. difficile* appears to be potentiated by antimicrobial therapy, particularly broad-spectrum β-lactams such as penicillins and cephalosporins. This was clearly illustrated in a clinical case of an adult rabbit developing fatal colitis after antibiotic treatment for *Pasteurella multocida* infection. The progression from antibiotic therapy to toxin-mediated colitis, confirmed by cytotoxicity assays and toxin neutralization with *C. difficile*, a specific antitoxin, strongly implicates antimicrobial-induced dysbiosis as a key predisposing factor for disease onset. In this context, antibiotics such as ampicillin and penicillin may disrupt normal gut flora, allowing germination and overgrowth of *C. difficile* spores, followed by toxin release and epithelial damage [20,27].

Histopathological observations from the previous literature further corroborate our findings, with typical lesions including fluid-filled ceca, mucosal necrosis, and heterophilic infiltration in the cecal lamina propria. In our own cases, similar postmortem lesions and fecal staining of the perineum were noted, suggestive of severe intestinal involvement. Notably, both human and animal studies have described a wide spectrum of lesion distribution, from mild enteritis to hemorrhagic typhlitis, often varying based on host species, age, and immune status [10,14,20,27].

Molecular comparisons have shown that animal strains of *C. difficile* often share genetic similarities with human epidemic ribotypes, raising the question of possible zoonotic transmission. The occurrence of toxigenic *C. difficile* in food-producing animals, including rabbits, reinforces the need to investigate their role as potential reservoirs of infection for humans, particularly in agricultural environments where biosecurity is limited [17,27].

Moreover, current findings challenge the assumption that *C. difficile*-associated diarrhea is a strictly nosocomial concern. Reports of community-acquired infections in humans and isolation of toxigenic strains from domestic and wild animals suggest a broader ecological presence for this pathogen [28,29]. In light of this, rabbits may serve as sentinels or reservoir species, especially given their known sensitivity to gut flora disturbances and frequent exposure to antimicrobials in farm settings [28,29].

Recent studies have further expanded the understanding of *C. difficile* epidemiology, virulence, and vaccine development, emphasizing its relevance beyond classical nosocomial settings and highlighting its role in both animal and human health. Emerging data also support the increasing diversity of circulating strains and their antimicrobial resistance patterns, reinforcing the importance of continuous surveillance and updated diagnostic approaches [30,31,32,33].

In addition, the lack of effective treatment once clinical signs appear, as documented both in our observations and in the literature, underscores the urgent need for preventive approaches. These may include improved management of antibiotic use, the development of effective vaccines based on local strains, and strict hygiene practices in commercial rabbitries [6,14,20,27].

The diagnostic interpretation of this case supports *C. difficile* as the primary etiological agent, based on consistent microbiological, toxigenic, and molecular findings, as well as the characteristic necrotizing intestinal lesions observed at necropsy. Differential diagnoses for fatal enteric disease in rabbits, including *Clostridium spiroforme*, *Escherichia coli*, and parasitic enteropathies such as coccidiosis, were considered. Parasitological examination, including flotation and Baermann techniques, yielded negative results, effectively excluding major parasitic causes. Although clinical signs such as diarrhea and rapid deterioration may overlap among these conditions, *C. difficile* infection is more commonly associated with antimicrobial-induced dysbiosis and toxin-mediated mucosal damage, as observed in the present case. In contrast, *C. spiroforme* is typically linked to iota toxin production, while coccidiosis is characterized by identifiable parasitic stages and specific intestinal lesions. Taken together, these findings support the etiological role of *C. difficile* and highlight the importance of integrated laboratory diagnostics in establishing an accurate diagnosis [6,22,23,34,35,36,37].

The novelty of this report lies in the integrated characterization of a toxigenic *C. difficile* strain isolated from both intestinal and extraintestinal sites, including bone marrow, suggesting systemic dissemination. Such findings are rarely documented in rabbits and contribute to expanding the current understanding of disease progression in this species. Additionally, the identification of a multidrug-resistant profile in this context provides clinically relevant information for antimicrobial stewardship in rabbit medicine.

The combined use of culture, toxin detection, and molecular confirmation provides a robust and reliable diagnostic framework for the identification of toxigenic *Clostridioides difficile*.

This study has several limitations. As a single-case report, the findings cannot be generalized to the wider rabbit population. Additionally, quantitative measurements such as organ weights were not recorded, and macroscopic images lacked scale references. Furthermore, advanced molecular typing techniques, such as ribotyping, were not performed, which limits the epidemiological characterization of the isolate.

Additional limitations include the absence of whole-genome sequencing, which prevented detailed epidemiological characterization and comparison with known zoonotic strains. Ante-mortem bacteremia confirmation and quantitative bacterial burden assessment were not performed. Furthermore, although aseptic sampling procedures were applied during necropsy, postmortem contamination of extraintestinal tissues cannot be entirely excluded.

## 4. Conclusions

This report provides novel evidence of possible systemic involvement and antimicrobial resistance in *C. difficile* infection in a rabbit, highlighting its clinical and epidemiological relevance. The isolation of a toxigenic strain from intestinal material, although rarely reported in lagomorphs, can occur under conditions of microbial dysbiosis and host susceptibility. Gross and histopathological findings, including necrotizing colitis, mucosal hemorrhages, and heterophilic infiltration, corroborate the role of *C. difficile* toxins in epithelial damage and severe gastrointestinal dysfunction. Moreover, the multidrug-resistant profile of the isolated strain underscores the risks associated with indiscriminate antibiotic use in companion and farmed rabbits, mirroring resistance patterns observed in human clinical isolates.

These observations reinforce the need for heightened vigilance regarding *C. difficile* in rabbit husbandry. Rabbits may act as reservoirs for toxigenic strains with zoonotic potential, given the genetic similarities between animal and human isolates, and the ecological presence of the pathogen outside of hospital settings. Preventive strategies, including judicious antimicrobial management, biosecurity measures, and development of effective vaccines, are essential to reduce the incidence and severity of infection.

## Figures and Tables

**Table 1:** Detection of *Clostridioides difficile* toxins A and B by ELISA.

Sample	OD Value	Cut-Off	Interpretation
Cecal content	1.25	0.35	Positive
Colon content	1.18	0.35	Positive

**Table 2:** Antimicrobial susceptibility profile of *Clostridioides difficile* isolate.

Antibiotic	MIC (µg/mL)	Interpretation
Clindamycin	>8	Resistant
Ampicillin	>16	Resistant
Tetracycline	>8	Resistant
Metronidazole	4	Intermediate
Vancomycin	0.5	Susceptible
Linezolid	1	Susceptible
Tigecycline	0.12	Susceptible

## Data Availability

The original contributions presented in this study are included in the article. Further inquiries can be directed to the corresponding author.
